# Supplementary material for: Genomic mosaicism with increased amyloid precursor protein (APP) gene copy number in single neurons from sporadic Alzheimer's disease brains
Source: eLife. 2015 Feb 4;4:e05116. doi: 10.7554/eLife.05116 (PMC4337608; doi:10.7554/eLife.05116)
Supplement: Figure 2—source data 1. — DOI: http://dx.doi.org/10.7554/eLife.05116.005 [file elife05116s001.docx]

Figure 2 – Source Data

| **Source Data: DNA Content Change Normalized to LYM** | | | | |
| --- | --- | --- | --- | --- |
|  | Sample | DI LYM | % Change | CV |
| AD CBL | 1252 | 1.101 | 10.086 | 5.14 |
| AD CBL | 1625 | 1.002 | 0.233 | 4.22 |
| AD CBL | 1861 | 0.920 | -7.975 | 5.92 |
| AD CBL | 1866 | 0.914 | -8.645 | 4.70 |
| AD CBL | 1868 | 0.944 | -5.556 | 4.41 |
| AD CBL | 1870 | 0.942 | -5.814 | 5.15 |
| AD CBL | 1875 | 0.947 | -5.344 | 4.16 |
| AD CBL | 1899 | 0.976 | -2.369 | 5.20 |
| AD CBL | 1912 | 0.956 | -4.431 | 6.72 |
| AD CBL | 1913 | 0.965 | -3.505 | 4.25 |
| AD CBL | 1916 | 0.957 | -4.345 | 7.42 |
| AD CBL | 1921 | 1.086 | 8.589 | 9.75 |
| AD CBL | 2401 | 1.020 | 2.038 | 3.70 |
| AD CBL | 2499 | 1.024 | 2.450 | 4.29 |
| AD CBL | 4199 | 1.044 | 4.358 | 3.11 |
| AD CBL | 102 | 1.185 | 18.460 | 4.57 |
|  | **N=16** | **Mean= 0.999** | **Mean=**  **-0.111** | **Mean= 5.168** |
|  |  |  |  |  |
| AD CTX | 102 | 1.161 | 16.139 | 9.42 |
| AD CTX | 268 | 1.094 | 9.389 | 10.10 |
| AD CTX | 736 | 1.098 | 9.765 | 8.66 |
| AD CTX | 1211 | 1.175 | 17.496 | 11.63 |
| AD CTX | 1252 | 1.214 | 21.391 | 10.60 |
| AD CTX | 1521 | 1.051 | 5.060 | 10.30 |
| AD CTX | 1562 | 1.159 | 15.924 | 10.50 |
| AD CTX | 1748 | 1.035 | 3.539 | 5.10 |
| AD CTX | 1861 | 1.057 | 5.729 | 9.83 |
| AD CTX | 1866 | 1.180 | 18.006 | 7.03 |
| AD CTX | 1868 | 1.021 | 2.113 | 9.53 |
| AD CTX | 1870 | 1.024 | 2.397 | 5.18 |
| AD CTX | 1875 | 1.016 | 1.609 | 7.26 |
| AD CTX | 1893 | 1.065 | 6.458 | 7.47 |
| AD CTX | 1899 | 1.072 | 7.188 | 7.58 |
| AD CTX | 1912 | 1.140 | 14.029 | 12.53 |
| AD CTX | 1913 | 1.017 | 1.691 | 6.10 |
| AD CTX | 1916 | 1.125 | 12.539 | 11.20 |
| AD CTX | 1921 | 1.204 | 20.388 | 10.43 |
| AD CTX | 2400 | 1.005 | 0.527 | 7.01 |
| AD CTX | 2401 | 1.045 | 4.473 | 6.64 |
| AD CTX | 2499 | 1.147 | 14.713 | 7.00 |
| AD CTX | 2500 | 1.050 | 5.023 | 8.51 |
| AD CTX | 4199 | 1.038 | 3.787 | 7.04 |
| AD CTX | 13173 | 1.098 | 9.789 | 7.17 |
| AD CTX | 30022 | 1.098 | 9.779 | 8.89 |
| AD CTX | 50341 | 1.081 | 8.059 | 6.76 |
| AD CTX | 60987 | 1.058 | 5.777 | 6.76 |
| AD CTX | 61788 | 1.007 | 0.725 | 6.82 |
| AD CTX | 62405 | 1.090 | 9.011 | 5.42 |
| AD CTX | 62439 | 1.002 | 0.196 | 9.43 |
| AD CTX | 62509 | 1.003 | 0.291 | 4.83 |
|  | **N=32** | **Mean= 1.082** | **Mean= 8.219** | **Mean=**  **8.209** |
|  |  |  |  |  |
| LYM | 29 | 1.047 | 4.725 | 2.41 |
| LYM | 83 | 0.960 | -3.989 | 2.44 |
| LYM | 187 | 0.945 | -5.513 | 2.23 |
| LYM | 1344 | 0.995 | -0.492 | 2.20 |
| LYM | 4519 | 0.986 | -1.394 | 5.69 |
| LYM | 4603 | 0.994 | -0.630 | 2.85 |
| LYM | 4609 | 1.012 | 1.167 | 2.76 |
| LYM | 4781 | 1.044 | 4.389 | 3.24 |
| LYM | 4801 | 0.968 | -3.220 | 4.06 |
| LYM | 4903 | 0.988 | -1.169 | 1.75 |
| LYM | 4984 | 1.003 | 0.286 | 2.98 |
| LYM | LYM | 0.975 | -2.513 | 2.55 |
| LYM | LYM 5269 | 1.025 | 2.513 | 3.91 |
| LYM | Lym 1 | 0.961 | -3.950 | 3.17 |
| LYM | Lym 1 | 1.003 | 0.317 | 2.85 |
| LYM | Lym 2 | 0.986 | -1.409 | 3.02 |
| LYM | Lym 3 | 0.996 | -0.422 | 3.25 |
| LYM | Lym 4 | 0.987 | -1.273 | 2.47 |
| LYM | Lym 5 | 1.037 | 3.747 | 3.61 |
| LYM | Lym 6 | 1.030 | 2.985 | 4.22 |
|  | **N= 20** | **Mean= 0.997** | **Mean=**  **-0.292** | **Mean= 3.083** |
|  |  |  |  |  |
| ND CBL | 299 | 0.944 | -5.605 | 4.81 |
| ND CBL | 827 | 1.074 | 7.410 | 3.78 |
| ND CBL | 1102 | 0.973 | -2.732 | 4.19 |
| ND CBL | 1344 | 0.980 | -2.017 | 5.70 |
| ND CBL | 1379 | 0.977 | -2.287 | 2.01 |
| ND CBL | 1471 | 0.963 | -3.670 | 5.98 |
| ND CBL | 1502 | 0.808 | -19.223 | 4.39 |
| ND CBL | 1569 | 0.985 | -1.461 | 2.21 |
| ND CBL | 1571 | 1.001 | 0.134 | 6.22 |
| ND CBL | 1901 | 1.003 | 0.278 | 4.85 |
| ND CBL | 2501 | 0.924 | -7.562 | 3.74 |
| ND CBL | 4546 | 0.964 | -3.571 | 2.49 |
|  | **N=12** | **Mean= 0.966** | **Mean=**  **-3.359** | **Mean= 4.196** |
|  |  |  |  |  |
| ND CTX | 299 | 1.007 | 0.703 | 5.37 |
| ND CTX | 318 | 1.021 | 2.059 | 4.50 |
| ND CTX | 389 | 1.017 | 1.687 | 5.07 |
| ND CTX | 719 | 1.082 | 8.207 | 6.90 |
| ND CTX | 827 | 1.050 | 4.983 | 6.45 |
| ND CTX | 946 | 1.048 | 4.834 | 7.04 |
| ND CTX | 955 | 1.063 | 6.299 | 6.93 |
| ND CTX | 1102 | 0.927 | -7.325 | 11.59 |
| ND CTX | 1301 | 0.991 | -0.892 | 3.34 |
| ND CTX | 1344 | 1.054 | 5.359 | 7.16 |
| ND CTX | 1379 | 1.074 | 7.353 | 6.81 |
| ND CTX | 1471 | 1.025 | 2.526 | 5.95 |
| ND CTX | 1502 | 0.910 | -8.952 | 8.48 |
| ND CTX | 1571 | 1.010 | 1.031 | 9.90 |
| ND CTX | 1901 | 1.040 | 3.957 | 7.51 |
| ND CTX | 2039 | 0.987 | -1.252 | 5.19 |
|  | **N= 36** | **Mean= 1.022** | **Mean= 2.238** | **Mean= 6.141** |

| ND CTX | 2501 | 0.918 | -8.166 | 6.31 |
| --- | --- | --- | --- | --- |
| ND CTX | 4238 | 1.030 | 3.015 | 5.98 |
| ND CTX | 4534 | 0.992 | -0.814 | 4.34 |
| ND CTX | 4546 | 1.068 | 6.847 | 7.64 |
| ND CTX | 11488 | 1.001 | 0.059 | 4.81 |
| ND CTX | 13188 | 1.107 | 10.683 | 8.86 |
| ND CTX | 13204 | 1.019 | 1.868 | 4.57 |
| ND CTX | 60329 | 1.008 | 0.810 | 4.56 |
| ND CTX | 60428 | 0.975 | -2.467 | 3.99 |
| ND CTX | 60524 | 1.033 | 3.346 | 6.52 |
| ND CTX | 60728 | 1.011 | 1.097 | 5.00 |
| ND CTX | 60772 | 1.018 | 1.817 | 5.93 |
| ND CTX | 60831 | 1.051 | 5.109 | 6.82 |
| ND CTX | 61218 | 1.049 | 4.886 | 6.73 |
| ND CTX | 61334 | 1.010 | 1.022 | 4.80 |
| ND CTX | 61545 | 1.065 | 6.453 | 7.63 |
| ND CTX | 62043 | 0.995 | -0.545 | 4.57 |
| ND CTX | PDC2 | 1.059 | 5.862 | 5.85 |
| ND CTX | PDC5 | 1.035 | 3.545 | 3.43 |
| ND CTX | PDC8 | 1.056 | 5.569 | 4.57 |

| **ANOVA for DNA Content change with Westra et al. Meta Data** | | | | |
| --- | --- | --- | --- | --- |
| Tukey's multiple comparisons test | Mean Diff. | 95% CI of diff. | Summary | Adjusted P Value |
| ND CBL vs. AD CBL | -3.247 | -9.016 to 2.521 | ns | 0.5253 |
| ND CBL vs. AD CTX | -11.58 | -16.69 to -6.464 | **** | < 0.0001 |
| ND CBL vs. ND CTX | -5.596 | -10.63 to -0.5615 | * | 0.0214 |
| ND CBL vs. LYM | -3.066 | -8.582 to 2.449 | ns | 0.5378 |
| AD CBL vs. AD CTX | -8.329 | -12.95 to -3.704 | **** | < 0.0001 |
| AD CBL vs. ND CTX | -2.349 | -6.887 to 2.189 | ns | 0.6063 |
| AD CBL vs. LYM | 0.1811 | -4.885 to 5.247 | ns | > 0.9999 |
| AD CTX vs. ND CTX | 5.98 | 2.310 to 9.650 | *** | 0.0001 |
| AD CTX vs. LYM | 8.51 | 4.205 to 12.82 | **** | < 0.0001 |
| ND CTX vs. LYM | 2.53 | -1.682 to 6.743 | ns | 0.4594 |
|  |  |  |  |  |
| ANOVA summary |  |  |  |  |
| F | 14.54 |  |  |  |
| P value | < 0.0001 |  |  |  |
| P value summary | **** |  |  |  |
| R square | 0.3438 |  |  |  |

| **ANOVA for DNA Content change from Current Study** | | | | |
| --- | --- | --- | --- | --- |
| Tukey's multiple comparisons test | Mean Diff. | 95% CI of diff. | Summary | Adjusted P Value |
|  |  |  |  |  |
| ND CBL vs. AD CBL | -2.131 | -8.992 to 4.729 | ns | 0.9089 |
| ND CBL vs. AD CTX | -10.46 | -16.58 to -4.339 | **** | < 0.0001 |
| ND CBL vs. ND CTX | -3.939 | -10.46 to 2.580 | ns | 0.4501 |
| ND CBL vs. LYM | -2.638 | -9.591 to 4.314 | ns | 0.8281 |
| AD CBL vs. AD CTX | -8.329 | -13.69 to -2.966 | *** | 0.0004 |
| AD CBL vs. ND CTX | -1.808 | -7.620 to 4.005 | ns | 0.9086 |
| AD CBL vs. LYM | -0.5071 | -6.802 to 5.788 | ns | 0.9994 |
| AD CTX vs. ND CTX | 6.522 | 1.603 to 11.44 | ** | 0.0034 |
| AD CTX vs. LYM | 7.822 | 2.341 to 13.30 | ** | 0.0013 |
| ND CTX vs. LYM | 1.301 | -4.621 to 7.222 | ns | 0.9729 |
|  |  |  |  |  |
| ANOVA summary |  |  |  |  |
| F | 9.254 |  |  |  |
| P value | < 0.0001 |  |  |  |
| P value summary | **** |  |  |  |
| R square | 0.2914 |  |  |  |

| **ANOVA For Coefficient of Variation Meta Data** | | | | |
| --- | --- | --- | --- | --- |
| Tukey's multiple comparisons test | Mean Diff. | 95% CI of diff. | Summary | Adjusted P Value |
|  |  |  |  |  |
| ND CBL vs. AD CBL | -0.9718 | -2.768 to 0.8248 | ns | 0.5646 |
| ND CBL vs. AD CTX | -4.013 | -5.606 to -2.421 | **** | < 0.0001 |
| ND CBL vs. ND CTX | -1.945 | -3.513 to -0.3765 | ** | 0.0072 |
| ND CBL vs. LYM | 1.113 | -0.6048 to 2.831 | ns | 0.3806 |
| AD CBL vs. AD CTX | -3.042 | -4.482 to -1.601 | **** | < 0.0001 |
| AD CBL vs. ND CTX | -0.9729 | -2.386 to 0.4407 | ns | 0.3189 |
| AD CBL vs. LYM | 2.085 | 0.5070 to 3.663 | ** | 0.0034 |
| AD CTX vs. ND CTX | 2.069 | 0.9256 to 3.212 | **** | < 0.0001 |
| AD CTX vs. LYM | 5.127 | 3.785 to 6.468 | **** | < 0.0001 |
| ND CTX vs. LYM | 3.058 | 1.746 to 4.370 | **** | < 0.0001 |
|  |  |  |  |  |
| ANOVA summary |  |  |  |  |
| F | 32.54 |  |  |  |
| P value | < 0.0001 |  |  |  |
| P value summary | **** |  |  |  |
| R square | 0.5397 |  |  |  |
