# Supplementary material for: Genomic mosaicism with increased amyloid precursor protein (APP) gene copy number in single neurons from sporadic Alzheimer's disease brains
Source: eLife. 2015 Feb 4;4:e05116. doi: 10.7554/eLife.05116 (PMC4337608; doi:10.7554/eLife.05116)
Supplement: Figure 5—source data 1. — DOI: http://dx.doi.org/10.7554/eLife.05116.011 [file elife05116s002.docx]

| **Figure 5 – Source Data: Raw dual point-paint probe FISH counts** | | | | | | | | | | | |
| --- | --- | --- | --- | --- | --- | --- | --- | --- | --- | --- | --- |
|  | **Total #** | **2**  **Copies** | **% Disomy** | **1**  **Copy** | **% Monosomy** | **3**  **Copies** | **% Trisomy** | **4**  **Copies** | **% Tetrasomy** | **Total Aneuploid** | **% Aneuploidy** |
| **1471 MFG** | 451 | 334 | 74.06 | 24 | 5.32 | 49 | 10.86 | 44 | 9.76 | 117 | 25.94 |
| **1916 CTX** | 458 | 362 | 79.04 | 12 | 2.58 | 49 | 10.70 | 35 | 7.64 | 96 | 20.92 |
| **1912 CTX** | 458 | 357 | 77.95 | 21 | 4.52 | 48 | 10.48 | 32 | 6.99 | 101 | 21.99 |
| **1875 CTX** | 703 | 564 | 80.23 | 23 | 3.28 | 73 | 10.38 | 30 | 4.27 | 126 | 17.93 |
| **1571 MFG** | 452 | 351 | 77.65 | 16 | 3.53 | 45 | 9.96 | 40 | 8.85 | 101 | 22.34 |
| **1861 CTX** | 820 | 645 | 78.66 | 15 | 1.81 | 78 | 9.51 | 70 | 8.54 | 163 | 19.86 |
| **713 MFG** | 768 | 630 | 82.03 | 14 | 1.84 | 67 | 8.72 | 47 | 6.12 | 128 | 16.68 |
| **1344 MFG** | 451 | 367 | 81.37 | 17 | 3.77 | 38 | 8.43 | 29 | 6.43 | 84 | 18.63 |
| **1866 CTX** | 456 | 392 | 85.96 | 13 | 2.82 | 32 | 7.02 | 19 | 4.17 | 64 | 14.01 |
| **1868 CTX** | 710 | 606 | 85.35 | 14 | 1.97 | 50 | 7.04 | 32 | 4.51 | 96 | 13.52 |
| **1870 CTX** | 450 | 394 | 87.56 | 14 | 3.12 | 23 | 5.11 | 19 | 4.22 | 56 | 12.45 |
| **827 MFG** | 454 | 386 | 85.02 | 17 | 3.72 | 30 | 6.61 | 21 | 4.63 | 68 | 14.96 |
| **1913 CTX** | 457 | 403 | 88.18 | 16 | 3.45 | 24 | 5.25 | 14 | 3.06 | 54 | 11.76 |
| **1921 CTX** | 462 | 400 | 86.58 | 22 | 4.86 | 22 | 4.76 | 17 | 3.68 | 61 | 13.30 |
