# Supplementary material for: Genomic mosaicism with increased amyloid precursor protein (APP) gene copy number in single neurons from sporadic Alzheimer's disease brains
Source: eLife. 2015 Feb 4;4:e05116. doi: 10.7554/eLife.05116 (PMC4337608; doi:10.7554/eLife.05116)
Supplement: Figure 6—source data 1. — DOI: http://dx.doi.org/10.7554/eLife.05116.014 [file elife05116s003.docx]

| **Figure 6 – Source Data: APP Exon 14 Statistics** | | | | |
| --- | --- | --- | --- | --- |
| Tukey's multiple comparisons test | Mean Diff. | 95% CI of diff. | Summary | Adjusted P Value |
| AD CBL vs. AD CTX | -1.064 | -1.985 to -0.1440 | * | 0.0163 |
| AD CBL vs. ND CBL | 0.4101 | -0.6466 to 1.467 | ns | 0.7449 |
| AD CBL vs. ND CTX | 0.898 | -0.2320 to 2.028 | ns | 0.1695 |
| AD CTX vs. ND CBL | 1.474 | 0.4426 to 2.506 | ** | 0.0016 |
| AD CTX vs. ND CTX | 1.962 | 0.8556 to 3.069 | **** | < 0.0001 |
| ND CBL vs. ND CTX | 0.4879 | -0.7345 to 1.710 | ns | 0.728 |
|  |  |  |  |  |
| ANOVA summary |  |  |  |  |
| F | 8.882 |  |  |  |
| P value | < 0.0001 |  |  |  |
| P value summary | **** |  |  |  |
| R square | 0.1509 |  |  |  |
|  |  |  |  |  |
|  | AD CBL | AD CTX | ND CBL | ND CTX |
| N | 46 | 52 | 31 | 25 |
| Mean | 2.338 | 3.402 | 1.928 | 1.44 |
| Median | 1.99 | 2.66 | 1.913 | 1.141 |
| Std. Deviation | 1.39 | 2.43 | 1.197 | 1.088 |
| Std. Error of Mean | 0.2049 | 0.337 | 0.215 | 0.2177 |
|  |  |  |  |  |

| **Figure 6 – Source Data: APP Exon 3 Statistics** | | | | |
| --- | --- | --- | --- | --- |
| Tukey's multiple comparisons test | Mean Diff. | 95% CI of diff. | Summary | Adjusted P Value |
|  |  |  |  |  |
| ND CBL vs. ND CTX | 0.6734 | -1.043 to 2.390 | ns | 0.7363 |
| ND CBL vs. AD CBL | 0.04305 | -1.288 to 1.374 | ns | 0.9998 |
| ND CBL vs. AD CTX | -1.522 | -2.822 to -0.2228 | * | 0.0147 |
| ND CTX vs. AD CBL | -0.6304 | -2.179 to 0.9180 | ns | 0.7133 |
| ND CTX vs. AD CTX | -2.196 | -3.717 to -0.6745 | ** | 0.0015 |
| AD CBL vs. AD CTX | -1.565 | -2.633 to -0.4982 | ** | 0.0012 |
|  |  |  |  |  |
| ANOVA summary |  |  |  |  |
| F | 7.821 |  |  |  |
| P value | < 0.0001 |  |  |  |
| P value summary | **** |  |  |  |
| R square | 0.1745 |  |  |  |
|  |  |  |  |  |
|  | ND CBL | ND CTX | AD CBL | AD CTX |
| Number of values | 20 | 13 | 38 | 44 |
| Mean | 2.277 | 1.604 | 2.234 | 3.8 |
| Median | 1.782 | 1.465 | 1.996 | 3.118 |
| Std. Deviation | 1.249 | 0.9229 | 1.096 | 2.617 |
| Std. Error of Mean | 0.2792 | 0.256 | 0.1779 | 0.3946 |
