# Supplementary material for: Genomic mosaicism with increased amyloid precursor protein (APP) gene copy number in single neurons from sporadic Alzheimer's disease brains
Source: eLife. 2015 Feb 4;4:e05116. doi: 10.7554/eLife.05116 (PMC4337608; doi:10.7554/eLife.05116)
Supplement: Figure 7—source data 1. — DOI: http://dx.doi.org/10.7554/eLife.05116.017 [file elife05116s004.docx]

Figure 7 – Soure Data

| Brain | Disease | % of Nuclei with Gain |
| --- | --- | --- |
| 1568 | ND | 20 |
| 1230 | ND | 25 |
| 1901 | ND | 21.7 |
| M233 | DS | 14.2 |
| 2500 | AD | 76.1 |
| 4199 | AD | 47.6 |
| 102 | AD | 63.2 |
| 2401 | AD | 38 |

ND Average: 22.24 +/- 1.47

AD Average: 55.78 +/- 8.535

P=.0216
